# Understanding Parkinson’s disease: current trends and its multifaceted complications

**DOI:** 10.3389/fnagi.2025.1617106

**Published:** 2025-09-18

**Authors:** Sameer A. Chaudhary, Sapana Chaudhary, Sakshi Rawat

**Affiliations:** RASA Life Science Informatics, Pune, India

**Keywords:** Parkinson’s disease (PD), neurodegenerative disorder, genetic and environmental factors, AI, molecular mechanisms bioinformatics

## Abstract

**Background:**

Parkinson’s disease (PD) is a multifactorial, progressive neurodegenerative disorder that primarily affects dopaminergic neurons in the substantia nigra. In addition to hallmark motor symptoms, it manifests a wide range of nonmotor complications, including cognitive decline, neuropsychiatric symptoms, autonomic dysfunction, and comorbid metabolic and infectious diseases.

**Objectives:**

This review aims to elucidate the molecular and cellular mechanisms underlying PD, explore the influence of genetic and environmental factors, evaluate current treatment limitations, and assess the clinical and socioeconomic burden globally. Emphasis is placed on emerging therapeutic avenues and innovative research directions.

**Methods:**

A structured literature review was conducted using PubMed, Scopus, and Web of Science databases. The search included articles published between 2010 and 2025, using keywords: “Parkinson’s disease,” “*α*-synuclein,” “dopaminergic degeneration,” “ferroptosis,” “deep brain stimulation,” “stem cell therapy,” and “AI in PD diagnosis.”

**Results:**

The review highlights a multifactorial etiology involving *α*-synuclein pathology, oxidative stress, mitochondrial dysfunction, genetic mutations (SNCA, LRRK2, VPS35), environmental toxins, and gut dysbiosis. Comorbidities such as HIV, diabetes, and cardiovascular disorders exacerbate disease burden. While Levodopa remains the gold standard, its limitations necessitate combination therapy and adjunct modalities such as deep brain stimulation and nanocarrier-based drug delivery. Emerging approaches—stem cell therapy, CRISPR-Cas9, and AI-enhanced diagnostics—show promise.

**Conclusion:**

PD management requires a paradigm shift toward precision medicine. Advancing research into biomarkers, immunotherapy, and systems biology, coupled with equitable access to care and early diagnosis tools, is critical to mitigating the global impact of PD.

## 1 Introduction

### 1.1 Literature review methodology

This review was conducted by searching PubMed, Scopus, and Google Scholar databases using the keywords “Parkinson’s disease,” “*α*-synuclein,” “dopaminergic neurons,” “LRRK2,” “mitochondrial dysfunction,” “comorbidities,” “levodopa,” and “neuroinflammation.” Studies published in English between 2000 and 2025 were included. Preference was given to peer-reviewed original research, clinical trials, and systematic reviews. Articles were excluded if they lacked relevance to neurobiological or clinical aspects of Parkinson’s disease. A total of 216 sources were shortlisted, of which 120 were cited based on scientific quality and thematic relevance.

Parkinson’s disease (PD) is the second most prevalent neurodegenerative disorder globally, surpassed only by Alzheimer’s disease (Zaguirre, 2023). Affecting more than 10 million people worldwide, its incidence is expected to double by 2040 due to population aging and improved survival rates (WHO, 2020; Zhong and Zhu, 2022). PD is clinically characterized by a triad of motor symptoms—bradykinesia, rigidity, and resting tremor—alongside a range of debilitating nonmotor symptoms such as depression, anxiety, cognitive decline, and autonomic dysfunction (Panicker et al., 2021; Hindle et al., 2018).

Parkinson’s disease (PD) poses an escalating global health challenge, with recent WHO data projecting a near doubling in cases by 2040 (WHO, 2020; Zhong and Zhu, 2022). The global societal burden is equally significant, with total direct and indirect costs estimated to exceed $112 billion USD annually by 2023 (Ola et al., 2022; Chaudhuri et al., 2024). Beyond the clinical manifestations, PD incurs heavy economic costs due to prolonged caregiving, disability, and lost productivity. High-income countries (HICs) typically provide structured care and insurance-based support, while low- and middle-income countries (LMICs) face limited access to neurologists, diagnostic tools, and long-term care facilities (Payami et al., 2023; Masoom et al., 2018). These global disparities necessitate region-specific policy planning, emphasizing both infrastructure and research capacity-building in under-resourced regions (Payami et al., 2023; Masoom et al., 2018).

Originally described in 1817 by James Parkinson as a purely motor disorder (Parkinson, 1817), PD is now recognized as a complex, multisystem disease involving a confluence of molecular, genetic, environmental, and immunological factors (Rode Karan Ganesh et al., 2024; Franco et al., 2021). Mutations in genes such as SNCA, LRRK2, PARK7, PINK1, and VPS35 are associated with familial forms of PD, while pesticide exposure, heavy metals, and industrial chemicals contribute to sporadic cases. Additionally, early nonmotor symptoms—such as anosmia, REM sleep behavior disorder (RBD), and gastrointestinal dysfunction—are increasingly identified as prodromal indicators.

PD poses a substantial economic burden globally. In the United States alone, the annual cost of PD exceeds $52 billion, including direct medical expenses and indirect costs such as lost productivity and caregiver burden (Ola et al., 2022; Chaudhuri et al., 2024). This burden is disproportionately heavier in low- and middle-income countries (LMICs), where underdiagnosis, limited therapeutic access, and healthcare disparities amplify disease impact (Payami et al., 2023; Masoom et al., 2018).

This review synthesizes contemporary insights into PD’s pathophysiology, genetic and environmental risk factors, comorbidities, and therapeutic innovations. Particular emphasis is placed on emerging tools such as stem cell therapy, CRISPR-Cas9 editing, AI-assisted diagnostics, and nanocarrier-mediated drug delivery. We also explore the role of global collaboration and policy strategies to address PD’s escalating prevalence and unmet clinical needs, especially in resource-limited settings.

## 2 Pathophysiology and molecular mechanisms of Parkinson’s disease

**Figure 1:**
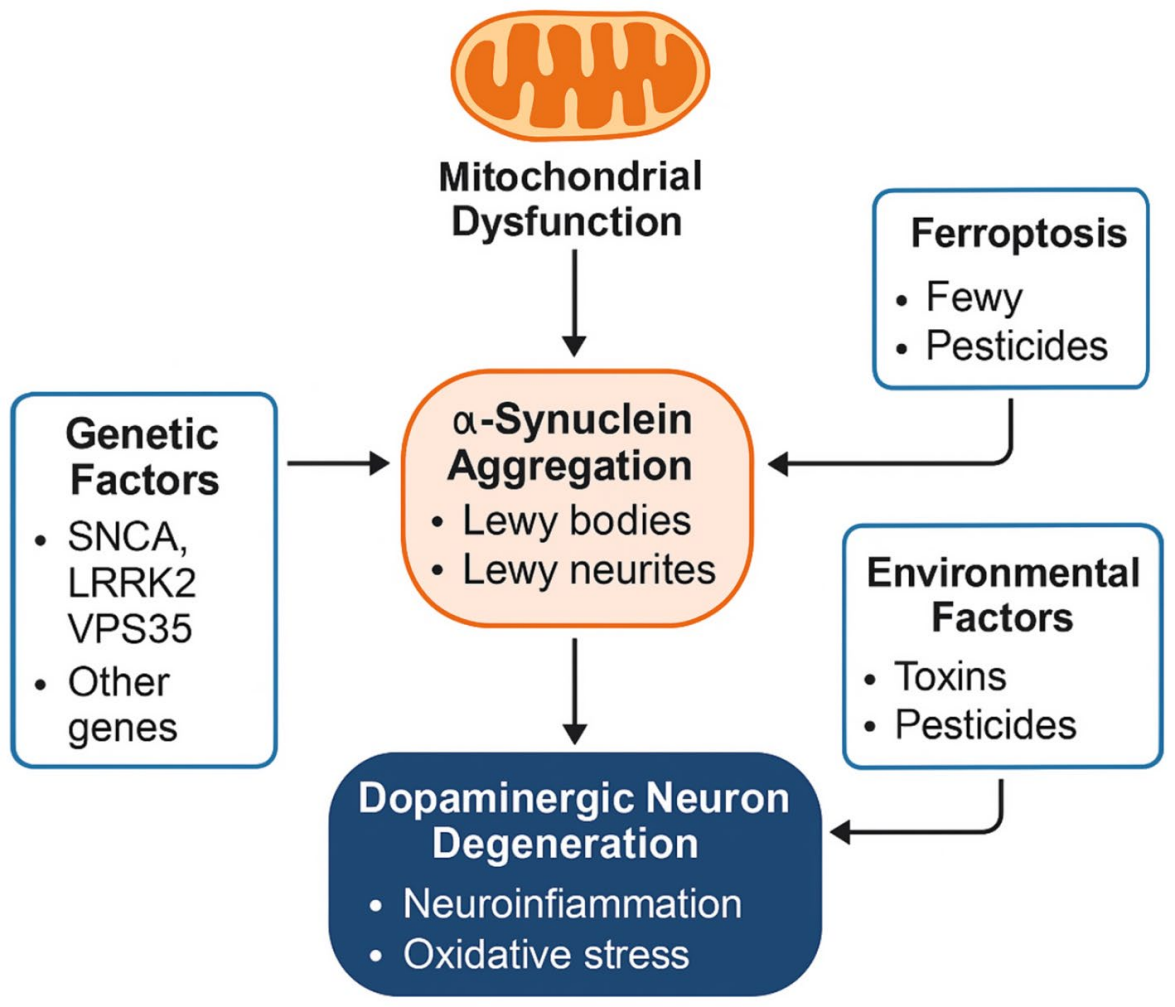
Molecular pathogenesis of Parkinson’s disease.

Parkinson’s disease (PD) is a multifactorial neurodegenerative disorder characterized by the progressive degeneration of dopaminergic neurons in the substantia nigra pars compacta (SNpc). A combination of protein misfolding, mitochondrial dysfunction, oxidative stress, neuroinflammation, and iron-mediated toxicity converge to disrupt neuronal homeostasis (Behl et al., 2022; Luo et al., 2022). This section outlines the key molecular events driving PD pathology (Figure 1).

This schematic illustrates the key molecular mechanisms contributing to dopaminergic neuron degeneration in Parkinson’s disease (PD). Genetic factors, including mutations in SNCA, LRRK2, and VPS35, promote *α*-synuclein aggregation into Lewy bodies and neurites. Mitochondrial dysfunction further enhances protein misfolding and oxidative stress. Environmental toxins and pesticides exacerbate the pathological process by triggering ferroptosis—a form of iron-dependent cell death—and contributing to synuclein aggregation. These converging pathways culminate in the progressive loss of dopaminergic neurons through neuroinflammation and oxidative stress.

### 2.1 *α*-synuclein aggregation and prion-like propagation

Recent findings support the hypothesis that α-synuclein pathology originates in the enteric nervous system and ascends to the brain via the vagus nerve. This aligns with the “body-first” PD subtype theory, suggesting that peripheral initiation may precede CNS involvement (Goedert et al., 2017; Wu et al., 2023). Moreover, exosomal transfer and templated seeding of misfolded *α*-synuclein contribute to its prion-like behavior (Dorsey et al., 2024). Lewy bodies, once thought to be mere markers, are now considered active participants in disease propagation due to their interaction with endolysosomal and mitochondrial pathways (Dorsey et al., 2024; Murphy and McKernan, 2022).

The misfolding and aggregation of *α*-synuclein into insoluble fibrils is a pathological hallmark of PD. These aggregates accumulate in neuronal cytoplasmic inclusions known as Lewy bodies and Lewy neurites (Goedert et al., 2017; Wu et al., 2023). Recent research supports a prion-like propagation model, where pathogenic α-synuclein spreads trans-neuronally via exosomes and synaptic transmission, initiating a chain reaction of misfolding across interconnected brain regions. This contributes to the progression from prodromal to symptomatic PD stages (Dorsey et al., 2024; Murphy and McKernan, 2022).

α-Synuclein interferes with synaptic vesicle trafficking, mitochondrial dynamics, and axonal transport, ultimately leading to synaptic dysfunction, impaired neurotransmission, and axonopathy. Post-translational modifications such as phosphorylation at Ser129 further enhance its aggregation propensity and neurotoxicity (Goedert et al., 2017; Wu et al., 2023).

In addition to complex I deficiency, dysfunction of mitochondrial quality control pathways—specifically PINK1/Parkin-mediated mitophagy—plays a pivotal role. Under physiological conditions, PINK1 accumulates on depolarized mitochondria and recruits Parkin to facilitate their degradation. Mutations in either gene impair this surveillance, allowing damaged mitochondria to persist and propagate oxidative stress (Franco et al., 2021; Luo et al., 2022). Furthermore, DJ-1, a redox-sensitive protein, is involved in protecting neurons from oxidative damage and modulating mitochondrial integrity, linking environmental and genetic risk (Franco et al., 2021; Luo et al., 2022).

### 2.2 Mitochondrial dysfunction and oxidative stress

Mitochondrial complex I deficiency is consistently observed in PD, leading to impaired ATP synthesis and overproduction of reactive oxygen species (ROS) (Chang and Chen, 2020; Zeng et al., 2024). Accumulated ROS cause oxidative damage to lipids, DNA, and proteins, creating a pro-degenerative cellular environment (Chang and Chen, 2020; Zeng et al., 2024). Genetic mutations in PINK1 and PARKIN, which regulate mitophagy, impair the clearance of damaged mitochondria, exacerbating oxidative stress (Franco et al., 2021; Luo et al., 2022).

Furthermore, LRRK2 mutations—particularly G2019S—are linked to disrupted mitochondrial dynamics, altered kinase activity, and aberrant autophagy (Franco et al., 2021; Luo et al., 2022). VPS35, a component of the retromer complex, contributes to mitochondrial fragmentation and impaired trafficking, particularly affecting dopaminergic neurons (Franco et al., 2021; Luo et al., 2022).

### 2.3 Ferroptosis: iron-mediated cell death

Ferroptosis is an emerging mechanism in PD, characterized by iron-dependent lipid peroxidation. Postmortem analyses reveal excessive iron accumulation in the SNpc, correlating with dopaminergic neuron loss (Wang et al., 2023; Xing et al., 2023). Iron promotes Fenton chemistry, enhancing hydroxyl radical production and driving oxidative injury (Wang et al., 2023; Xing et al., 2023). Key regulators such as glutathione peroxidase 4 (GPX4), ferritin, and transferrin are dysregulated in PD, suggesting therapeutic potential for ferroptosis inhibitors and iron chelators (Wang et al., 2023; Xing et al., 2023).

Studies show that *α*-synuclein may bind iron directly, promoting its aggregation, while lipid peroxidation products further stabilize toxic oligomers (Wang et al., 2023; Xing et al., 2023).

### 2.4 Neuroinflammation and cytokine activation

Chronic neuroinflammation is another driver of PD progression. Activated microglia and astrocytes release proinflammatory cytokines including tumor necrosis factor-alpha (TNF-*α*), interleukin-6 (IL-6), and interleukin-1β (IL-1β), creating a hostile neuroimmune milieu (Isik et al., 2023; Eidson et al., 2017; Fu et al., 2023). These cytokines disrupt blood–brain barrier integrity, potentiate oxidative stress, and induce apoptotic signaling pathways (Isik et al., 2023; Eidson et al., 2017; Fu et al., 2023).

In PD brains, persistent glial activation is associated with regions of dopaminergic degeneration, indicating a spatial correlation (Isik et al., 2023; Eidson et al., 2017; Fu et al., 2023). In addition, peripheral inflammation—linked to comorbid conditions like diabetes and HIV—may exacerbate central neuroinflammatory responses (Isik et al., 2023; Eidson et al., 2017; Fu et al., 2023).

Epigenetic regulation also influences proteostasis in PD. Alterations in histone acetylation and DNA methylation affect the transcription of key autophagy genes such as ATG5 and LC3B. MicroRNAs (miRNAs) like miR-34b/c and miR-7 have been implicated in regulating *α*-synuclein expression and lysosomal biogenesis. These findings suggest that targeting epigenetic modifiers and miRNA networks may offer novel therapeutic opportunities (Isik et al., 2023; Eidson et al., 2017; Fu et al., 2023).

### 2.5 Disrupted proteostasis and autophagy-lysosomal dysfunction

The autophagy-lysosomal pathway (ALP) and ubiquitin-proteasome system (UPS) are critical for degrading misfolded proteins. In PD, mutations in GBA, ATP13A2, and PARK9 impair lysosomal function, leading to α-synuclein accumulation and cellular toxicity (Behl et al., 2022; Luo et al., 2022). Dysregulated mTOR signaling and defective chaperone-mediated autophagy further inhibit proteostasis (Behl et al., 2022; Luo et al., 2022).

In particular, LRRK2 mutations impair endolysosomal trafficking, contributing to α-synuclein clearance failure and promoting neurodegeneration (Behl et al., 2022; Luo et al., 2022).

## 3 Epidemiology and global burden of Parkinson’s disease

Parkinson’s disease (PD) is one of the fastest-growing neurological disorders globally, driven primarily by population aging, environmental exposures, and improved longevity. According to the Global Burden of Disease (GBD) Study 2019, the number of individuals living with PD more than doubled from 2.5 million in 1990 to over 6.1 million in 2016, with projections exceeding 12 million by 2040 (Zhong and Zhu, 2022). Age-standardized prevalence and disability-adjusted life years (DALYs) associated with PD have shown a 156% increase between 1990 and 2019 (Zhong and Zhu, 2022).

### 3.1 Age and sex distribution

**Figure 2:**
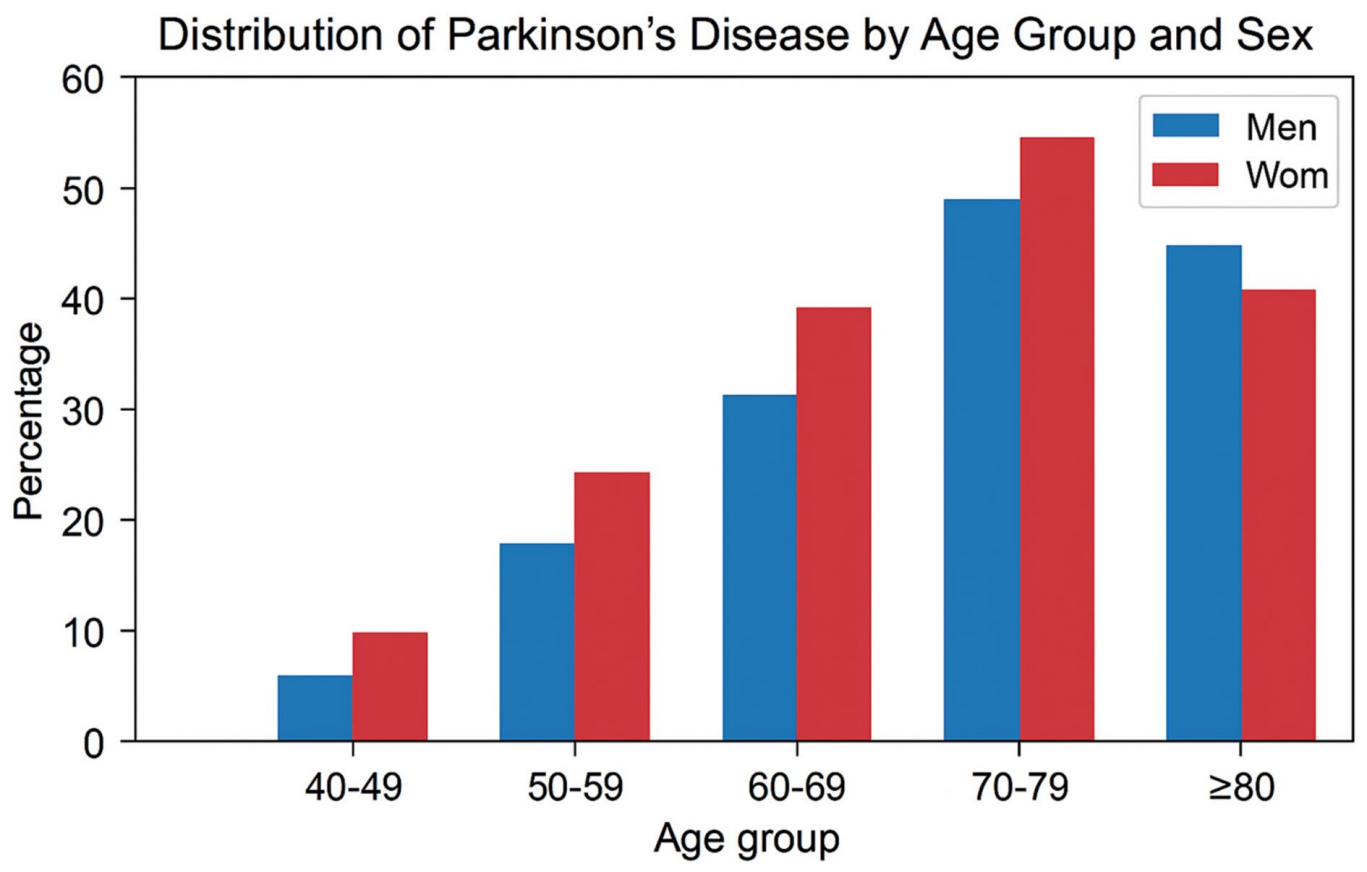
Distribution of Parkinson’s disease by age group and sex. This bar graph illustrates the percentage distribution of Parkinson’s disease across different age groups (40–49, 50–59, 60–69, 70–79, and ≥80 years) for men (blue bars) and women (red bars). The prevalence increases with age in both sexes, peaking in the 70–79 age group. Women show a higher percentage than men in the 40–79 age groups, with the trend reversing slightly in the ≥80 group. Data suggest a sex-specific and age-related trend in Parkinson’s disease prevalence.

PD predominantly affects individuals over the age of 60, with incidence rising sharply after the age of 70. Epidemiological analyses reveal a male predominance, with men exhibiting approximately 1.5 times higher risk than women (Ben-Shlomo et al., 2024). This disparity is hypothesized to arise from differences in estrogen-mediated neuroprotection, lifestyle factors, and occupational exposure to neurotoxins (Ben-Shlomo et al., 2024) (Figure 2).

### 3.2 Geographical variations

**Figure 3:**
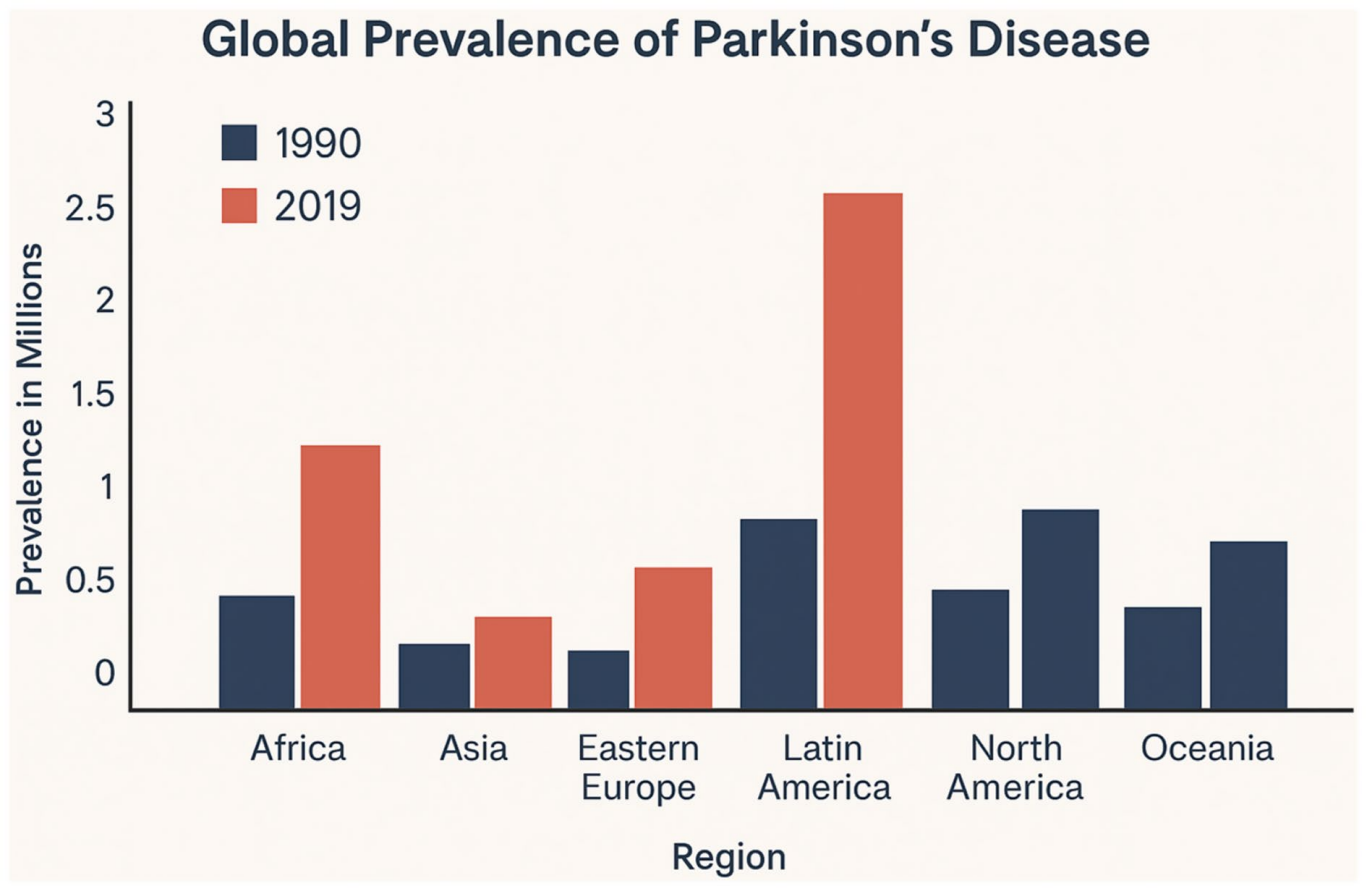
Global prevalence of Parkinson’s disease (1990–2019). This bar chart compares the regional prevalence of Parkinson’s disease across six global regions—Africa, Asia, Eastern Europe, Latin America, North America, and Oceania—between 1990 and 2019. All regions show marked increases in PD prevalence, with the most significant rise observed in Latin America and Africa. These trends reflect population aging, improved diagnosis, and environmental exposure patterns in both high-income and low- and middle-income countries.

The prevalence and incidence of PD vary across countries and regions, influenced by demographic structure, industrialization, access to healthcare, and awareness levels. High-income countries (HICs) report higher prevalence rates due to better diagnostic tools, registry systems, and longer life expectancy. However, low- and middle-income countries (LMICs) are experiencing the steepest rise in new PD cases, largely due to demographic transition and under-recognition in the past (Masoom et al., 2018; Rahman et al., 2025) (Figure 3).

### 3.3 Socioeconomic and health system impact

PD imposes a significant socioeconomic burden, including direct healthcare costs (e.g., medications, hospitalizations, and surgery) and indirect costs (e.g., lost income, caregiver absenteeism, and reduced productivity). In the United States alone, the economic burden of PD exceeded $52 billion in 2020, with caregivers losing an average of $19,000 in annual income due to reduced work hours or job loss (Chaudhuri et al., 2024).

**Figure 4:**
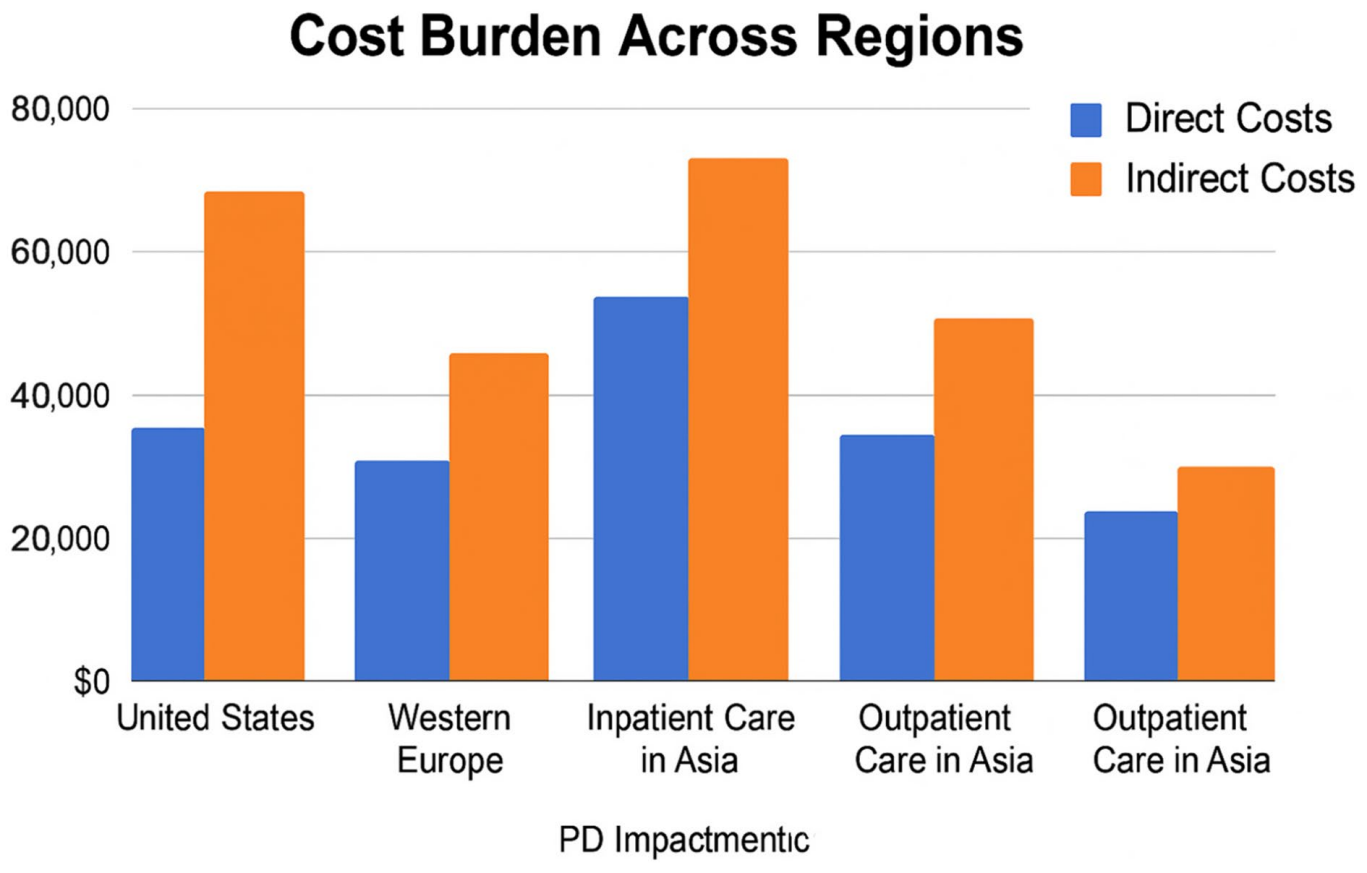
Cost burden across regions and care sectors in Parkinson’s disease. This grouped bar chart compares direct and indirect costs of PD across healthcare systems, including the United States, Western Europe, and inpatient/outpatient care in Asia. Indirect costs—such as lost productivity and caregiver burden—surpass direct medical expenses in most settings. The figure underscores the need for cost-effective, system-wide policy responses to support PD care globally.

**Figure 5:**
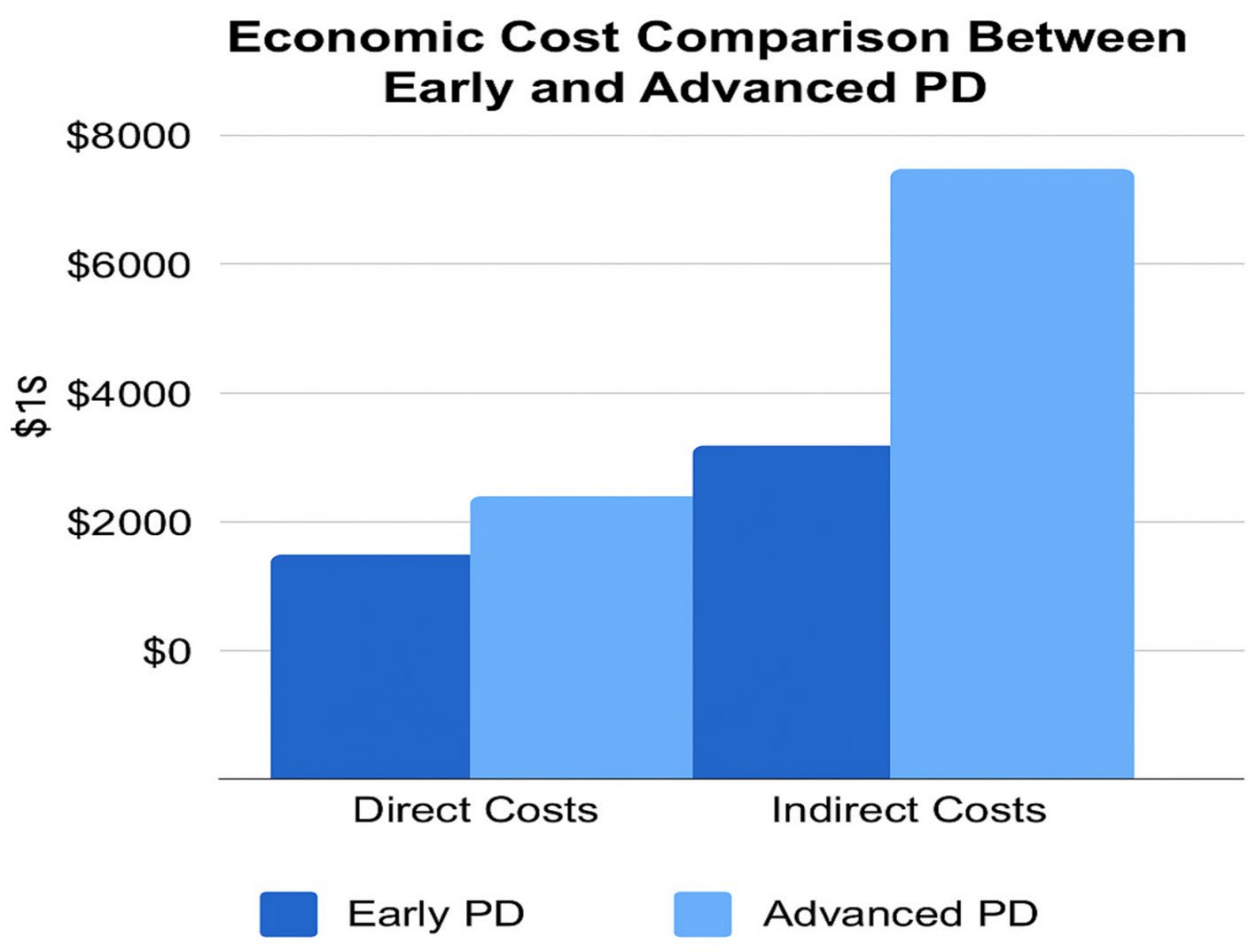
Economic cost breakdown between direct and indirect components by PD stage. This side-by-side bar chart compares the direct and indirect costs of early versus advanced PD. While both cost categories rise with disease progression, indirect costs such as caregiver absenteeism, job loss, and social support account for the greatest burden in advanced stages. This figure supports the need for integrated financial support and early intervention frameworks.

Comparative studies show that while HICs absorb higher per-patient costs through insurance systems and assistive infrastructure, LMICs face a disproportionately higher household-level burden, where out-of-pocket expenses can consume over 50% of monthly income (Ola et al., 2022) (Figures 4, 5).

### 3.4 Barriers to diagnosis and registry development

A significant proportion of PD patients in LMICs remain undiagnosed or misdiagnosed, especially in rural and low-resource settings. Contributing factors include:

- Lack of trained neurologists or movement disorder clinics.
- Cultural stigma and poor health literacy.
- Overlapping symptomatology with aging and other comorbid conditions.

Establishing national PD registries, integrating PD into noncommunicable disease (NCD) strategies, and investing in mobile health screening tools are critical steps toward improved surveillance and resource allocation. (Gupta and Dhawan, 2021).

## 4 PD complications and comorbidities

Recent epidemiological surveys have refined our understanding of comorbidity patterns in PD. For example, depression and anxiety occur in up to 60% of patients, while dementia develops in nearly 80% of advanced cases (Panicker et al., 2021; Leta et al., 2022; Lee et al., 2022; Elefante et al., 2021). Emerging research also suggests that cardiovascular dysfunction—especially orthostatic hypotension and cardiac denervation—contributes to early mortality (Grosu et al., 2023). HIV and T2DM are increasingly recognized not just as co-existing conditions but as synergistic factors that worsen neurodegeneration (Denaro et al., 2022; Hassan et al., 2020; Hassan et al., 2020; Camargo et al., 2019). These conditions may not only complicate treatment but also precede motor onset, underscoring their diagnostic and prognostic value.

Parkinson’s disease (PD) is increasingly recognized as a systemic, multisystem disorder that encompasses a wide array of nonmotor complications and comorbidities. These features often precede motor symptoms and can serve as prodromal indicators, profoundly influencing diagnosis, treatment response, and quality of life. The multifaceted nature of PD comorbidities necessitates a precision medicine approach tailored to the patient’s full clinical context (Panicker et al., 2021; Leta et al., 2022).

### 4.1 Neuropsychiatric symptoms: depression, anxiety, and cognitive decline

Over 40–60% of PD patients experience depression and anxiety, which are often underdiagnosed and misattributed to motor disability (Lee et al., 2022; Elefante et al., 2021). These symptoms are linked to degeneration in serotonergic and noradrenergic circuits, particularly in the raphe nuclei and locus coeruleus, and may manifest years before classical motor signs (Lee et al., 2022; Elefante et al., 2021).

Cognitive impairment, ranging from mild cognitive dysfunction to Parkinson’s disease dementia (PDD), affects up to 80% of individuals in advanced stages (25). This is attributed to Lewy body pathology in cortical regions, cholinergic depletion, and altered frontostriatal connectivity. Psychiatric symptoms may also be exacerbated by dopamine replacement therapy (DRT), particularly dopamine agonists, which can trigger impulse control disorders, hallucinations, and psychosis (Burchill et al., 2024; Taslim et al., 2024).

Diagnostic challenge: Differentiating neuropsychiatric manifestations from motor-induced cognitive slowness or medication side effects requires multidisciplinary assessment. Use of validated scales (e.g., MoCA, GDS, HADS) in routine care is recommended.

### 4.2 Cardiovascular and autonomic dysfunction

Autonomic dysfunction is a hallmark nonmotor feature of PD and includes:

- Orthostatic hypotension (in up to 30–40%).
- Arrhythmias, bradycardia, and QT prolongation.
- Urinary incontinence, constipation, and sexual dysfunction.

Cardiac sympathetic denervation, detectable via ^123^I-MIBG scintigraphy, is strongly associated with disease progression (Grosu et al., 2023). These cardiovascular abnormalities increase fall risk, exacerbate fatigue, and limit the use of medications such as anticholinergics and MAO-B inhibitors (Grosu et al., 2023).

Management complications: Cardiovascular symptoms complicate pharmacotherapy by interacting with anti-parkinsonian drugs that modulate autonomic tone, requiring dose balancing and often necessitating cardiology consultation (Sharkey and Modarai, 2018; Al-Kuraishy et al., 2023).

### 4.3 HIV co-infection and antiretroviral therapy

HIV-positive individuals are at increased risk of parkinsonism due to basal ganglia vulnerability to viral neurotoxicity, chronic inflammation, and mitochondrial stress (Denaro et al., 2022; Hassan et al., 2020). HIV-associated neurocognitive disorders (HAND) share overlapping features with PD, including bradykinesia and cognitive dysfunction (Denaro et al., 2022; Hassan et al., 2020).

Moreover, antiretroviral therapy (ART), particularly protease inhibitors and nucleoside reverse transcriptase inhibitors, can:

- Interfere with dopamine metabolism.
- Alter pharmacokinetics of Levodopa.
- Exacerbate oxidative stress and mitochondrial dysfunction (Marin et al., 2021; Ferrazzoli et al., 2016).

Thus, HIV–PD co-management requires careful drug selection and neurology–infectious disease collaboration to avoid worsening symptoms or drug–drug interactions.

### 4.4 Type 2 diabetes mellitus (T2DM)

T2DM is a significant comorbidity in PD, with shared pathogenic features including:

- Insulin resistance in the CNS.
- Mitochondrial dysfunction.
- Chronic low-grade inflammation.
- Advanced glycation end products (AGEs) that impair dopaminergic signaling.

Epidemiological studies suggest a 30–50% increased risk of PD in patients with T2DM (Hassan et al., 2020; Camargo et al., 2019). T2DM may also accelerate PD progression, worsen cognitive decline, and impair motor response to Levodopa. Conversely, certain GLP-1 receptor agonists, such as exenatide, have shown neuroprotective effects in clinical trials, indicating the potential for cross-therapeutic benefits (Troshneva and Ametov, 2022).

### 4.5 Gut dysbiosis and the microbiota-gut-brain axis

Emerging evidence highlights the role of gut microbiota in PD pathophysiology. PD patients frequently exhibit dysbiosis, characterized by:

- Reduced short-chain fatty acid–producing bacteria (e.g., *Faecalibacterium*).
- Increased pro-inflammatory genera (e.g., *Enterobacteriaceae*).

Gut inflammation and barrier dysfunction may facilitate *α*-synuclein misfolding in the enteric nervous system, with retrograde propagation via the vagus nerve (Li et al., 2023; Turco et al., 2023). Animal models support this bottom-up mechanism of disease initiation (Li et al., 2023; Turco et al., 2023).

Probiotics, prebiotics, and synbiotics are under clinical investigation as adjunct therapies to modulate gut-brain interactions and reduce systemic inflammation (Chan et al., 2022; Hashish and Salama, 2023).

## 5 Treatment challenges and therapeutic innovations

Despite being the cornerstone of symptomatic management, current Parkinson’s disease (PD) treatments are palliative rather than disease-modifying. Levodopa remains the most effective agent for controlling motor symptoms, but its long-term use leads to motor complications, reduced efficacy, and treatment resistance (Kulisevsky, 2022; Naqvi et al., 2020), highlighting the need for combination strategies and novel delivery platforms (Kulisevsky, 2022; Naqvi et al., 2020). This section evaluates major therapeutic modalities, limitations, and emerging innovations.

### 5.1 Levodopa and the rationale for combination therapy

Levodopa, often co-administered with carbidopa to inhibit peripheral metabolism, is the gold standard for PD treatment. However, chronic use is associated with:

- Motor fluctuations (wearing-off, on–off phenomena).
- Dyskinesia.
- Diminished response in advanced stages (Kulisevsky, 2022; Naqvi et al., 2020).

To enhance efficacy and reduce complications, combination therapies employ:

- MAO-B inhibitors (e.g., rasagiline).
- Dopamine agonists (e.g., pramipexole).
- COMT inhibitors (e.g., entacapone) (Suárez Castro et al., 2016; Khan et al., 2023).

Yet these agents often exacerbate neuropsychiatric and cardiovascular side effects, especially in older patients, limiting their use (Suárez Castro et al., 2016; Khan et al., 2023).

### 5.2 Deep brain stimulation (DBS) vs. pharmacological treatment

DBS is indicated in patients with severe motor fluctuations and Levodopa-induced dyskinesia. It involves stereotactic implantation of electrodes into deep brain structures, most commonly:

- Subthalamic nucleus (STN).
- Globus pallidus internus (GPi) (Niethammer et al., 2017).

*Efficacy comparison*:

DBS significantly improves motor function and quality of life, particularly in patients with advanced disease and medication-refractory symptoms (Hacker et al., 2020; Rawls, 2022; Hitti et al., 2019; Rahimpour et al., 2022). Compared to pharmacological treatment, DBS provides sustained symptom control and reduces Levodopa requirements. However, risks include surgical complications, hemorrhage, infection, and neuropsychiatric side effects, necessitating careful patient selection and multidisciplinary evaluation (Cury et al., 2022; Wagle Shukla et al., 2017) (Table 1).

**Table 1:** Efficacy comparison of Levodopa and DBS across clinical parameters including symptoms, side effects, and cost.

Parameter	Levodopa	DBS
Motor symptom control	High	High
Nonmotor symptom improvement	Limited	Variable
Disease modification	None	None
Side effects	Dyskinesia, fluctuations	Surgical risks, cognition
Cost and accessibility	Low–moderate	High (surgical + device)

### 5.3 Overcoming the blood–brain barrier (BBB): nanocarrier-based drug delivery

The BBB severely restricts CNS penetration of most therapeutic agents. Emerging nanocarrier platforms aim to improve drug bioavailability and target specificity (Naqvi et al., 2020; Inamdar et al., 2024):

- *Liposomes*: biocompatible vesicles for encapsulating Levodopa and neuroprotective agents.
- *Polymeric nanoparticles*: controlled release systems (e.g., PLGA-based).
- *Dendrimers and micelles*: facilitate passage via receptor-mediated transcytosis.
- *Exosome-inspired nanocarriers*: for endogenous biomimicry and enhanced biocompatibility.

These carriers can be engineered for surface modification with ligands targeting dopaminergic neurons or inflamed brain regions, enabling precision delivery and minimizing off-target toxicity (Naqvi et al., 2020; Inamdar et al., 2024).

### 5.4 Gene therapy: targeting underlying molecular defects

Gene therapy in PD aims to:

- Restore dopamine synthesis: e.g., via delivery of aromatic L-amino acid decarboxylase (AADC).
- Enhance neuronal survival: e.g., delivery of GDNF or neurturin genes.
- Silence or correct mutations: e.g., *SNCA* and *LRRK2* via RNA interference (RNAi) or CRISPR-Cas9 (Niethammer et al., 2017; Sen et al., 2025., Inoue et al., 2023; Sharma et al., 2025).

AAV2-based vectors have been used in clinical trials to deliver therapeutic genes to the striatum or SNpc, showing safety and moderate benefit (Niethammer et al., 2017; Sen et al., 2025). However, challenges remain in target specificity, long-term expression, and immune response (Inoue et al., 2023; Sharma et al., 2025).

### 5.5 Stem cell-based approaches

Stem cell therapies offer the potential to replace lost dopaminergic neurons and restore neural circuitry. Promising cell types include:

- Induced pluripotent stem cells (iPSCs): Autologous and genetically matched.
- Embryonic stem cell–derived DA progenitors.
- Mesenchymal stem cells (MSCs): For immunomodulation and trophic support (Gholamzad et al., 2023; Madrazo et al., 2019).

Preclinical models and early-phase trials have shown motor improvement and graft survival, but issues such as immune rejection, tumorigenicity, and ethical concerns need resolution (Xiao and Tan, 2023; Li et al., 2023; Cha et al., 2023; Barker, 2019).

### 5.6 CRISPR-Cas9 and genomic correction

CRISPR-Cas9 enables precise genome editing and holds transformative potential for monogenic PD, particularly:

- Correction of *LRRK2 G2019S*, *SNCA* triplications, or *PINK1* mutations.
- Creation of isogenic cellular models for drug screening (Inoue et al., 2023; Sharma et al., 2025).

Currently, CRISPR is limited to *in vitro* and animal studies due to safety and ethical barriers, but advances in delivery vectors and base editing technologies may accelerate clinical translation (Inoue et al., 2023; Sharma et al., 2025).

### 5.7 Artificial intelligence (AI) in diagnosis and monitoring

AI and machine learning (ML) are revolutionizing PD management through:

- *Early diagnosis*: analyzing digital biomarkers (e.g., voice, gait, handwriting).
- *Disease progression models*: integrating longitudinal clinical and imaging data.
- *Treatment response prediction*: personalizing drug regimens based on patient profiles (Burchill et al., 2024; Taslim et al., 2024).

**Figure 6:**
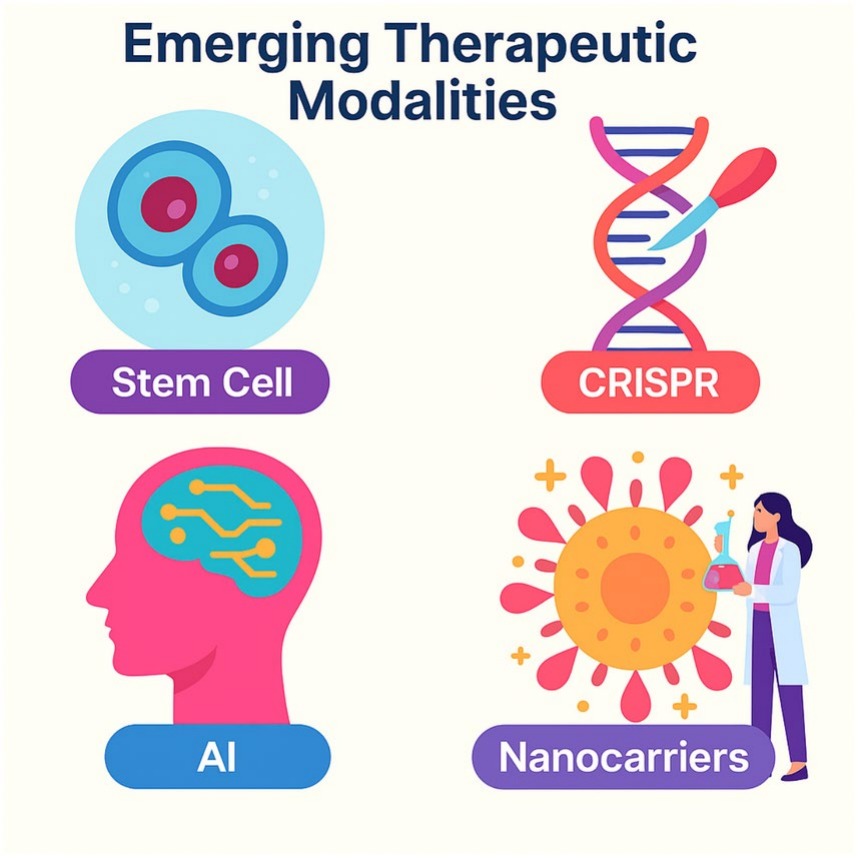
Emerging therapeutic modalities in Parkinson’s disease. This infographic highlights four cutting-edge therapeutic strategies under investigation for Parkinson’s disease (PD). Stem cell therapy aims to replace lost dopaminergic neurons using induced pluripotent stem cells or embryonic-derived progenitors. CRISPR-Cas9 gene editing enables precise correction of PD-associated mutations such as *LRRK2* or *SNCA*. Artificial Intelligence (AI) supports early diagnosis, disease monitoring, and personalized treatment planning through machine learning algorithms analyzing clinical, wearable, and omics data. Nanocarriers, including liposomes and polymeric nanoparticles, are engineered to cross the blood–brain barrier for targeted drug delivery and enhanced bioavailability of neuroprotective compounds.

Wearable sensors and smartphones now enable real-time symptom tracking, and ML algorithms can stratify patients for adaptive trial designs and biomarker discovery (Burchill et al., 2024; Taslim et al., 2024) (Figure 6).

## 6 Future projections and the need for research

Despite significant strides in symptomatic therapy, Parkinson’s disease (PD) remains incompletely understood and incurable, with no current treatment that halts or reverses neurodegeneration. A strategic shift is needed from reactive management to preventive and disease-modifying paradigms.

Future research must address three critical gaps:

- *Early diagnosis*—through robust biomarkers and AI-enabled screening.
- *Personalized therapeutics*—integrating genomics, imaging, and digital phenotyping.
- *Equitable care models*—ensuring access to diagnosis, treatment, and trials across all income settings.

These goals will require interdisciplinary collaboration, investment in longitudinal registries, and ethical frameworks for the deployment of novel technologies like gene editing and neural interfaces.

### 6.1 Biomarker discovery and validation

Biomarkers are essential for diagnosing PD in its prodromal phase, tracking progression, and predicting treatment response. Promising classes include:

- *Protein biomarkers*: *α*-synuclein (total and phosphorylated), neurofilament light chain (NfL), DJ-1.
- *Inflammatory markers*: TNF-α, IL-6, IL-1β in serum/CSF.
- *Lipid peroxidation products*: for ferroptosis monitoring.
- *Imaging biomarkers*: dopaminergic transporter (DaT) SPECT, PET tracers for α-synuclein.
- *Microbiome profiles*: gut microbial shifts linked to motor and nonmotor symptomatology.

Longitudinal cohort initiatives like the Parkinson’s Progression Markers Initiative (PPMI) and BioFIND are accelerating multi-omic biomarker integration. However, most candidates require standardization across labs, ethnic groups, and disease stages before clinical deployment.

### 6.2 Innovation in clinical trial design

Traditional PD trials are constrained by:

- Slow progression of disease markers.
- Symptomatic overlap.
- High inter-patient variability.

Future trials should adopt:

- *Adaptive designs*: allow protocol adjustments based on interim data.
- *Basket trials*: group patients by molecular subtype (e.g., *LRRK2+*, *SNCA+*, idiopathic).
- *Decentralized trials*: leverage digital tools and wearables for home-based data capture.
- *Multi-arm, multi-stage (MAMS) protocols*: increase efficiency in assessing multiple interventions.

Moreover, digital endpoints (e.g., gait variability, voice modulation, tremor amplitude) using AI analytics offer objective and continuous monitoring over traditional scales like the Unified Parkinson’s Disease Rating Scale (UPDRS).

### 6.3 Advancing precision medicine

The heterogeneity of PD underscores the need for personalized therapy. Key enablers include:

- *Genetic profiling*: stratifying patients by mutations (*LRRK2*, *GBA*, *PINK1*) to match targeted treatments.
- *AI-driven treatment selection*: using machine learning models trained on clinical, genomic, and imaging data to guide drug choice and dosage.
- *Multi-omics integration*: transcriptomics, epigenomics, proteomics, and metabolomics to define subtypes and therapeutic windows.

Precision medicine is already guiding trials for LRRK2 inhibitors, GCase modulators, and GLP-1 analogues, representing a shift toward therapies tailored to molecular etiology and patient-specific risk profiles.

### 6.4 Addressing global and socioeconomic gaps

Most PD research and trials are concentrated in high-income countries, neglecting ethnic diversity, LMIC-specific risk factors, and accessibility challenges. Priorities include:

- *Building local research capacity*: training neurologists and establishing centers of excellence in LMICs.
- *Developing affordable, scalable tools*: e.g., mobile-based screening apps, low-cost wearable diagnostics.
- *Engaging underserved populations in trials*: to ensure global applicability of therapies and biomarkers.

Public-private partnerships and South–South collaborations are essential to ensure global equity in PD care and innovation.

### 6.5 Research priorities at a glance

See Table 2.

**Table 2:** Emerging research priorities and recommended actions in Parkinson’s disease (PD) management.

Research focus	Recommended action
Biomarker development	Validate CSF/plasma α-synuclein, NfL, cytokines across populations
AI in PD management	Expand tools for early detection and individualized therapy
Ferroptosis pathway	Investigate iron chelators and lipid peroxidation inhibitors
Microbiome–brain axis	Develop microbiota-targeted interventions (e.g., synbiotics)
Stem cell and gene therapies	Advance iPSC-based trials and AAV-mediated gene editing
Clinical trial design	Implement adaptive, decentralized, and biomarker-enriched trials

## 7 Conclusion and policy recommendations

Parkinson’s disease (PD) represents a mounting global challenge, characterized by rising prevalence, incomplete understanding of disease mechanisms, and inadequate access to care—especially in resource-limited regions. Although symptomatic therapies like Levodopa and Deep Brain Stimulation (DBS) offer short-term relief, they fail to alter disease progression or address the wide array of nonmotor symptoms and comorbidities.

The evolution of PD management now depends on:

- Precision diagnostics to detect disease earlier and differentiate subtypes.
- Molecular-targeted interventions that go beyond dopamine replacement.
- Health system reforms that integrate PD care into national strategies, particularly in low- and middle-income countries (LMICs).

Global research equity, digital innovation, and collaborative policy action are essential to build a future where PD prevention, care, and cure are accessible to all.

### 7.1 Summary of key insights

- *Pathogenesis*: PD is driven by interlinked mechanisms including *α*-synuclein aggregation, mitochondrial dysfunction, ferroptosis, and neuroinflammation.
- *Genetic and environmental interplay*: mutations in *SNCA*, *LRRK2*, *PINK1*, and *VPS35* interact with toxins and metabolic stressors to trigger degeneration.
- *Comorbidities*: neuropsychiatric symptoms, HIV, T2DM, cardiovascular dysfunction, and gut dysbiosis significantly modify disease trajectory and complicate treatment.
- *Therapeutic limitations*: levodopa loses efficacy over time; DBS is invasive and inaccessible to many; the blood–brain barrier (BBB) remains a major hurdle in CNS drug delivery.
- *Emerging strategies*: gene therapy, stem cells, nanocarriers, and AI-driven diagnostics show promise but require further validation, regulatory clarity, and equitable access.
- *Economic and regional disparities*: LMICs experience disproportionate diagnostic delays, treatment inaccessibility, and caregiver burden.

### 7.2 Actionable research recommendations

See Table 3.

**Table 3:** Actionable research priorities and proposed interventions for advancing Parkinson’s disease treatment.

Priority Area	Proposed action
Biomarker development	Fund longitudinal, multi-omics studies including LMIC populations
Clinical trial innovation	Adopt adaptive, digital, and basket trial models
AI/ML for personalized care	Deploy AI to stratify patients and predict therapy response
Gene and cell-based therapies	Support scalable iPSC and CRISPR-based preclinical and early clinical studies
Ferroptosis and neuroinflammation	Investigate iron chelation and anti-cytokine interventions
Gut microbiota modulation	Advance trials on synbiotic, probiotic therapies

### 7.3 Health system and policy recommendations

1. Expand early screening and AI-based diagnostic tools in primary and rural care settings to detect PD in prodromal stages.
2. Subsidize essential therapies, including Levodopa combinations and deep brain stimulation (DBS), via public–private partnerships.
3. Establish national PD registries and surveillance systems, integrated into broader NCD frameworks.
4. Train a multidisciplinary PD care workforce, including neuropsychiatrists, geriatricians, rehabilitation specialists, and community health workers.
5. Mandate inclusion of LMIC patients in international clinical trials to ensure therapeutic relevance and accessibility across populations.
6. Promote open-access data sharing platforms (e.g., PPMI, ASAP) and South–South scientific collaboration.

### 7.4 Vision forward

The future of PD care must be multidimensional, digitally enabled, and globally inclusive. Investments in biomarker discovery, personalized therapies, and innovative trial frameworks should be matched by equitable health system reforms and coordinated policy alignment. Only through interdisciplinary collaboration, open science, and technology-driven democratization of care can the field transition from symptomatic palliation to genuine disease modification (Table 4).

**Table 4:** Common comorbidities in Parkinson’s disease, their prevalence, underlying mechanisms, and clinical implications.

Comorbidity	Prevalence in PD (%)	Pathophysiological link	Clinical implication
Depression and anxiety	40–60%	Serotonergic and noradrenergic dysfunction; neuroinflammation	Reduced quality of life; may precede motor symptoms
Cognitive decline	Up to 80%	Lewy pathology in cortex; cholinergic loss	Progresses to dementia; affects treatment planning
Cardiovascular dysfunction	30–50%	Autonomic failure; α-synuclein in cardiac nerves	Orthostatic hypotension; arrhythmias; fall risk
Type 2 diabetes mellitus	30–50% comorbidity	Insulin resistance; mitochondrial stress; inflammation	Accelerates PD progression; impairs Levodopa response
HIV co-infection	Higher in endemic regions	Mitochondrial stress; viral toxicity; ART interactions	Overlap with neurocognitive symptoms; drug interactions

## References

[ref1] Al-kuraishy H. M. Al-Gareeb A. I. Alexiou A. Papadakis M. Alsayegh A. A. Almohmadi N. H.. (2023). Pros and cons for statins use and risk of Parkinson's disease: an updated perspective. Pharmacol. Res. Perspect. 11:e01063. doi: 10.1002/prp2.1063, PMID: 36811160 PMC9944858

[ref3] Barker R. A. (2019). Designing stem-cell-based dopamine cell replacement trials for Parkinson's disease. Nat. Med. 25, 1045–1053. doi: 10.1038/s41591-019-0507-2, PMID: 31263283

[ref4] Behl T. Madaan P. Sehgal A. Singh S. Anwer M. K. Makeen H. A.. (2022). Mechanistic insights expatiating the redox-active-metal-mediated neuronal degeneration in Parkinson’s disease. Int. J. Mol. Sci. 23:678. doi: 10.3390/ijms23020678, PMID: 35054862 PMC8776156

[ref5] Ben-Shlomo Y. Darweesh S. Llibre-Guerra J. Marras C. San Luciano M. Tanner C. (2024). The epidemiology of Parkinson's disease. Lancet 403, 283–292. doi: 10.1016/S0140-6736(23)01419-8, PMID: 38245248 PMC11123577

[ref6] Burchill E. Watson C. J. Fanshawe J. B. Badenoch J. B. Rengasamy E. Ghanem D. A.. (2024). The impact of psychiatric comorbidity on Parkinson's disease outcomes: a systematic review and meta-analysis. Lancet Reg. Health - Eur. 39:100870. doi: 10.1016/j.lanepe.2024.100870, PMID: 38361749 PMC10867667

[ref7] Camargo F. David M. Alzira F. deSiqueira Carvalho A. (2019). Analysis of the relationship between type II diabetes mellitus and Parkinson's disease: a systematic review. Parkinson's Dis. 2019:4951379. doi: 10.1155/2019/4951379 31871617 PMC6906831

[ref8] Cha Y. Park T.-Y. Leblanc P. Kim K.-S. (2023). Current status and future perspectives on stem cell-based therapies for Parkinson's disease. J. Mov. Disorders 16, 22–41. doi: 10.14802/jmd.22141, PMID: 36628428 PMC9978267

[ref9] Chan D. G. Ventura K. Villeneuve A. Du Bois P. Holahan M. R. (2022). Exploring the connection between the gut microbiome and Parkinson's disease symptom progression and pathology: implications for supplementary treatment options. J. Parkinsons Dis. 12, 2339–2352. doi: 10.3233/JPD-223461, PMID: 36278360 PMC9837702

[ref10] Chang K.-H. Chen C.-M. (2020). The role of oxidative stress in Parkinson’s disease. Antioxidants 9:597. doi: 10.3390/antiox9070597, PMID: 32650609 PMC7402083

[ref11] Chaudhuri K. R. Azulay J. P. Odin P. Lindvall S. Domingos J. Alobaidi A.. (2024). Economic burden of Parkinson's disease: a multinational, real-world, cost-of-illness study. Drugs Real World Outcomes 11, 1–11. doi: 10.1007/s40801-023-00410-1, PMID: 38193999 PMC10928026

[ref12] Cury R. G. Pavese N. Aziz T. Z. Krauss J. K. Moro E. (2022). Gaps and roadmap of novel neuromodulation targets for treatment of gait in Parkinson’s disease. NPJ Parkinsons Dis. 8:8. doi: 10.1038/s41531-022-00222-9 35017551 PMC8752758

[ref13] Denaro F. Worthington M. Williams S. Benedetti F. Zella D. Davis H.. (2022). Identification of increased blood brain barrier permeability in the substantia nigra of the HIV-1 transgenic rat. Microsc. Microanal. 28, 3214–3215. doi: 10.1017/S1431927622011953

[ref14] Dorsey E. R. De Miranda B. R. Horsager J. Borghammer P. (2024). The body, the brain, the environment, and Parkinson’s disease. J. Parkinsons Dis. 14, 363–381. doi: 10.3233/JPD-240019, PMID: 38607765 PMC11091648

[ref15] Eidson L. N. Kannarkat G. T. Barnum C. J. Chang J. Chung J. Caspell-Garcia C.. (2017). Candidate inflammatory biomarkers display unique relationships with alpha-synuclein and correlate with measures of disease severity in subjects with Parkinson’s disease. J. Neuroinflammation 14:164. doi: 10.1186/s12974-017-0935-1, PMID: 28821274 PMC5563061

[ref16] Elefante C. Brancati G. E. Bacciardi S. Mazzucchi S. Del Prete E. Palermo G.. (2021). Prevalence and clinical correlates of comorbid anxiety and panic disorders in patients with Parkinson's disease. J. Clin. Med. 10:2302. doi: 10.3390/jcm10112302, PMID: 34070549 PMC8198165

[ref17] Ferrazzoli D. Carter A. Ustun F. S. Palamara G. Ortelli P. Maestri R.. (2016). Dopamine replacement therapy, learning and reward prediction in Parkinson's disease: implications for rehabilitation. Front. Behav. Neurosci. 10:121. doi: 10.3389/fnbeh.2016.00121, PMID: 27378872 PMC4906006

[ref18] Franco R. Rivas-Santisteban R. Navarro G. Pinna A. Reye I. (2021). Genes implicated in familial Parkinson’s disease provide a dual picture of nigral dopaminergic neurodegeneration with mitochondria taking center stage. Int. J. Mol. Sci. 22:4643. doi: 10.3390/ijms22094643, PMID: 33924963 PMC8124903

[ref19] Fu J. Chen S. Liu J. Yang J. Ou R. Zhang L.. (2023). Serum inflammatory cytokines levels and the correlation analyses in Parkinson’s disease. Front. Cell Dev. Biol. 11:1104393. doi: 10.3389/fcell.2023.1104393, PMID: 36875766 PMC9978777

[ref20] Gholamzad A. Sadeghi H. Azizabadi Farahani M. Faraji A. Rostami M. Khonche S.. (2023). Neural stem cell therapies: promising treatments for neurodegenerative diseases. Neurol. Lett. 2, 55–68. doi: 10.61186/nl.2.2.55

[ref21] Goedert M. Jakes R. Spillantini M. G. (2017). The synucleinopathies: twenty years on. J. Parkinsons Dis. 7, S51–S69. doi: 10.3233/JPD-179005, PMID: 28282814 PMC5345650

[ref22] Grosu A. I. Ambarus C. Bajko Z. Motoc A. (2023). Parkinson's disease and cardiovascular involvement: edifying insights. Biomed. Rep. 18:16074. doi: 10.3892/br.2023.16074 PMC9944619 36846617

[ref23] Gupta B. M. Dhawan S. M. (2021). Parkinson’s disease research by India: a scientometric assessment of publications output for the period 1990-2019. J. Brain Neurosci. 5:017.

[ref24] Hacker M. L. Turchan M. Heusinkveld L. E. Currie A. D. Millan S. H. Molinari A. L.. (2020). Deep brain stimulation in early-stage Parkinson disease: five-year outcomes. Neurology 95, e393–e401. doi: 10.1212/WNL.0000000000009946, PMID: 32601120 PMC7455319

[ref25] Hashish S. Salama M. (2023). The role of an altered gut microbiome in Parkinson’s disease: a narrative review. Appl. Microbiol. 3, 429–447. doi: 10.3390/applmicrobiol3020030

[ref26] Hassan A. Sharma Kandel R. Mishra R. Gautam J. Alaref A. Jahan N. (2020). Diabetes mellitus and Parkinson's disease: shared pathophysiological links and possible therapeutic implications. Cureus 12:e9853. doi: 10.7759/cureus.9853, PMID: 32832307 PMC7437092

[ref27] Hindle J. V.. (2018). Cognitive impairment in Parkinson’s disease: current challenges and future prospects. NPJ Parkinsons Dis. 4:19. doi: 10.1038/s41531-018-0055-3 29951580 PMC6018742

[ref28] Hitti F. L. Ramayya A. G. McShane B. J. Yang A. I. Vaughan K. A. Baltuch G. H. (2019). Long-term outcomes following deep brain stimulation for Parkinson's disease. J. Neurosurg. 132, 205–210. doi: 10.3171/2018.8.JNS182081 30660117

[ref29] Inamdar A. Gurupadayya B. Gautam M. Sharma A. Pathak R. Sharma H. (2024). Ai-driven innovations in assessing stress, anxiety, and mental health. Curr. Psychiatry Res. Rev. 21, 1–28. doi: 10.2174/0126660822334997241216062002

[ref30] Inoue S. Nishimura K. Gima S. Nakano M. Takata K. (2023). CRISPR-Cas9-edited SNCA knockout human induced pluripotent stem cell-derived dopaminergic neurons and their vulnerability to neurotoxicity. Biol. Pharm. Bull. 46, 517–522. doi: 10.1248/bpb.b22-00839, PMID: 36858582

[ref31] Isik S. Yeman Kiyak B. Akbayir R. Seyhali R. Arpaci T. (2023). Microglia mediated neuroinflammation in Parkinson’s disease. Cells 12:1012. doi: 10.3390/cells12071012, PMID: 37048085 PMC10093562

[ref32] Khan S. Agnihotri J. Patil S. Khan N. (2023). Drug repurposing: a futuristic approach in drug discovery. J. Pharm. Biol. Sci. 11, 66–69. doi: 10.18231/j.jpbs.2023.011

[ref33] Kulisevsky J. (2022). Pharmacological management of Parkinson's disease motor symptoms: update and recommendations from an expert. Tratamiento farmacológico de los síntomas motores de la enfermedad de Parkinson: actualización y recomendaciones de un experto. Rev. Neurol. 75, S1–S10. doi: 10.33588/rn.75s04.2022217 PMC10281635 36342310

[ref34] Lee Y. Chang Y. Y. Chen Y. F. Lin T. K. Hung C. F. Chiou Y. J.. (2022). Prevalence and risk factors of depression between patients with Parkinson's disease and their caregivers: a one-year prospective study. Healthcare 10:1305. doi: 10.3390/healthcare10071305, PMID: 35885832 PMC9318994

[ref35] Leta V. Urso D. Batzu L. Lau Y. Mathew D. Boura I.. (2022). Viruses, parkinsonism and Parkinson’s disease: the past, present and future. J. Neural Transm. 129, 1119–1132. doi: 10.1007/s00702-022-02536-y 36036863 PMC9422946

[ref36] Li F. Ji J. Xue J. Schweitzer J. Song B. (2023). Ethical and safety considerations in stem cell-based therapy for Parkinson’s disease. London: IntechOpen.

[ref37] Luo Q. Sun W. Wang Y.-F. Li J. Li D.-W. (2022). Association of p53 with neurodegeneration in Parkinson's disease. Parkinson's Disease 2022:6600944. doi: 10.1155/2022/6600944, PMID: 35601652 PMC9117072

[ref38] Madrazo I. Kopyov O. Ávila-Rodríguez M. A. Ostrosky F. Carrasco H. Kopyov A.. (2019). Transplantation of human neural progenitor cells (NPC) into putamina of parkinsonian patients: a case series study, safety and efficacy four years after surgery. Cell Transplant. 28, 269–285. doi: 10.1177/0963689718820271, PMID: 30574805 PMC6425108

[ref39] Marin R. C. Behl T. Negrut N. Bungau S. (2021). Management of Antiretroviral Therapy with boosted protease inhibitors-Darunavir/ritonavir or Darunavir/Cobicistat. Biomedicines 9:313. doi: 10.3390/biomedicines9030313, PMID: 33803812 PMC8003312

[ref40] Masoom M. Abbas Z. Xu L. (2018). Epidemiology of Parkinson's disease—east versus west. Mov. Disord. Clin. Pract. doi: 10.1002/MDC3.12568 PMC6174379 30363342

[ref41] Murphy J. McKernan D. P. (2022). The effect of aggregated alpha synuclein on synaptic and axonal proteins in Parkinson's disease—a systematic review. Biomol. Ther. 12:1199. doi: 10.3390/biom12091199, PMID: 36139038 PMC9496556

[ref42] Naqvi S. Panghal A. Flora S. J. S. (2020). Nanotechnology: a promising approach for delivery of neuroprotective drugs. Front. Neurosci. 14:494. doi: 10.3389/fnins.2020.00494, PMID: 32581676 PMC7297271

[ref43] Niethammer M. Tang C. C. LeWitt P. A. Rezai A. R. Leehey M. A. Ojemann S. G.. (2017). Long-term follow-up of a randomized AAV2-GAD gene therapy trial for Parkinson’s disease. JCI Insight 2:e90133. doi: 10.1172/jci.insight.90133, PMID: 28405611 PMC5374069

[ref44] Ola V. Puri I. Goswami D. Vibha D. Shukla G. Goyal V.. (2022). Annual cost of Care of Parkinson's disease and its determinants in North India - a cost of illness study with patient perspective. Ann. Indian Acad. Neurol. 25, 660–663. doi: 10.4103/aian.aian_779_21, PMID: 36211186 PMC9540931

[ref45] Panicker N. Ge P. Dawson V. L. Dawson T. M. (2021). The cell biology of Parkinson's disease. J. Cell Biol. 220:e202012095. doi: 10.1083/jcb.202012095, PMID: 33749710 PMC8103423

[ref46] Parkinson J. (1817). An essay on the shaking palsy. J. Neuropsychiatry Clin. Neurosci. 14, 223–236. doi: 10.1176/jnp.14.2.223 11983801

[ref47] Payami H. Cohen G. Murchison C. F. Sampson T. R. Standaert D. G. Wallen Z. D. (2023). Population fraction of Parkinson's disease attributable to preventable risk factors. NPJ Parkinsons Dis. 9:159. doi: 10.1038/s41531-023-00603-z, PMID: 38052871 PMC10698155

[ref48] Rahimpour S. Zhang S.-C. Vitek J. L. Mitchell K. T. Turner D. A. (2022). Comparative efficacy of surgical approaches to disease modification in Parkinson disease. NPJ Parkinson's Disease 8:33. doi: 10.1038/s41531-022-00296-w, PMID: 35338165 PMC8956588

[ref49] Rahman M. Wang Z. Ou Z. Pan J. Tang S. Duan D.. (2025). Global trends in the incidence, prevalence, and years lived with disability of Parkinson's disease in 204 countries/territories from 1990 to 2019. Front. Pub. Health 9:776847. doi: 10.3389/fpubh.2021.776847 PMC8688697 34950630

[ref50] Rawls A. E. (2022). Surgical therapies for Parkinson disease. Continuum 28, 1301–1313. doi: 10.1212/CON.0000000000001160, PMID: 36222766

[ref51] Rode Karan Ganesh M. Jadhav V. Sanap G. (2024). Parkinson disease. IJRASET 12:57956. doi: 10.22214/ijraset.2024.57956

[ref52] Sen A. Sarkar K. Pathak R. Sharma H. Al N. A. Sharma P. D.. (2025). Unravelling LRRK2 pathways in Parkinson’s disease: mechanisms and intricacies. Curr. Signal Transduct. Ther. 20, 1–10. doi: 10.2174/0115743624360618250507112532

[ref2] Sharkey A. R. Modarai B. (2018). Medical management of risk factors for vascular disease. Surgery. 36, 265–271. doi: 10.1016/J.MPSUR.2018.03.007

[ref53] Sharma H. Binte Ibrahim S. Al Noman A. Zohora U. F. T. Shifa F. A. Siddika S.. (2025). The potential of coenzyme Q10 in Alzheimer’s disease: reducing IL-17 induced inflammation and oxidative stress for neuroprotection. Curr. Drug Res. Rev. 17, 1–14. doi: 10.2174/0125899775373406250411104442, PMID: 40277120

[ref55] Suárez Castro E. Santos-García D. de Deus Fonticoba T. Expósito Ruíz I. Tuñas Gesto C. Macías Arribí M. (2016). Causes and factors related to dopamine agonist withdrawal in Parkinson’s disease. Brain Behav. 6:e00453. doi: 10.1002/brb3.453, PMID: 27247848 PMC4864043

[ref56] Taslim S. Shadmani S. Saleem A. Saleem A. R. Kumar A. Brahma F.. (2024). Neuropsychiatric disorders: bridging the gap between neurology and psychiatry. Cureus 16:51655. doi: 10.7759/cureus.51655, PMID: 38313968 PMC10838116

[ref57] Troshneva A. Y. Ametov A. S. (2022). Bolezn' Parkinsona i sakharnyi diabet 2-go tipa: svyaz' mekhanizmov patogeneza i obshchie terapevticheskie podkhody Parkinson's disease and type 2 diabetes mellitus: interrelation of pathogenetic mechanisms and general therapeutic approaches. Zh. Nevrol. Psikhiatr. Im. S.S. Korsakova 122, 12–18. doi: 10.17116/jnevro202212211212 36412150

[ref58] Turco L. Opallo N. Buommino E. De Caro C. Pirozzi C. Mattace Raso G.. (2023). Zooming into gut dysbiosis in Parkinson’s disease: new insights from functional mapping. Int. J. Mol. Sci. 24:9777. doi: 10.3390/ijms24119777, PMID: 37298727 PMC10253733

[ref59] Wagle Shukla A. Zeilman P. Fernandez H. Bajwa J. A. Mehanna R. (2017). DBS programming: an evolving approach for patients with Parkinson's disease. Parkinson's Disease 2017:8492619. doi: 10.1155/2017/8492619, PMID: 29147598 PMC5632902

[ref60] Wang P. Chen Q. Tang Z. Wang L. Gong B. Li M.. (2023). Uncovering ferroptosis in Parkinson’s disease via bioinformatics and machine learning, and reversed deducing potential therapeutic natural products. Front. Genet. 14:1231707. doi: 10.3389/fgene.2023.1231707, PMID: 37485340 PMC10358855

[ref61] WHO (2020). World Health Organization. 2020. Deaths from Parkinson's disease dataset. Our World in Data. Global Health Estimates. Geneva: WHO.

[ref62] Wu S. Hernandez Villegas N. C. Schekman R. (2023). Chemical disaggregation of alpha-synuclein fibrils as a therapy for synucleinopathies. Proc. Natl. Acad. Sci. 120:e2300965120. doi: 10.1073/pnas.2300965120, PMID: 36888654 PMC10242719

[ref63] Xiao B. Tan E.-K. (2023). Cell replacement for Parkinson’s disease: advances and challenges. Neural Regen. Res. 18, 2693–2694. doi: 10.4103/1673-5374.373710, PMID: 37449626 PMC10358655

[ref64] Xing N. Dong Z. Wu Q. Zhang Y. Kan P. Han Y.. (2023). Identification of ferroptosis related biomarkers and immune infiltration in Parkinson’s disease by integrated bioinformatic analysis. BMC Med. Genet. 16:1493. doi: 10.1186/s12920-023-01493-2 PMC10012699 36918862

[ref65] Zaguirre N. J. V. (2023). Development of electrospun fibers for drug delivery (Bachelor’s thesis). Barcelona: Universitat Politècnica de Catalunya.

[ref66] Zeng Z. Cen Y. Xiong L. Hong G. Luo Y. Luo X. (2024). Dietary copper intake and risk of Parkinson’s disease: a cross-sectional study. Biol. Trace Elem. Res. 202, 955–964. doi: 10.1007/s12011-023-03750-9, PMID: 37462848 PMC10803382

[ref67] Zhong Q. Q. Zhu F. (2022). Trends in prevalence cases and disability-adjusted life-years of Parkinson's disease: findings from the global burden of disease study 2019. Neuroepidemiology 56, 261–270. doi: 10.1159/000524208, PMID: 35320800

